# Data on water consumption in streptozotocin-induced diabetic mice by a novel peach gum-derived polysaccharide

**DOI:** 10.1016/j.dib.2017.04.022

**Published:** 2017-04-23

**Authors:** Yuting Wang, Dingbo Lin, Xiaoli Wang, Wei Zhu, Junli Ye, Guohuai Li, Zhaocheng Ma, Xiuxin Deng

**Affiliations:** a Key Laboratory of Horticultural Plant Biology, Ministry of Education, Huazhong Agricultural University, Wuhan 430070, China; b Department of Nutritional Sciences, Oklahoma State University, 301 Human Sciences, Stillwater, OK 74078, USA

**Keywords:** Peach gum, Polysaccharide, Diabetic, Water consumption

## Abstract

The data presented in this article are related to the article entitled “The impact of a novel peach gum-derived polysaccharide on postprandial blood glucose control in streptozotocin-induced diabetic mice” (Wang et al., 2017) [1]. Polydipsia was one of the most important symptoms of diabetic mellitus (DM) mice, which showed more water consumption than normal ones. The water consumption of DM mice in different groups administrated with metformin hydrochloride or a novel polysaccharide (coded as PGPSD) were exhibited in this article (Fig. 1). The field data set is made publicly available to enable critical or extended analyzes.

**Specifications Table**

**Table:** 

Subject area	Biology
More specific subject area	Anti-diabetic
Type of data	Figure
How data was acquired	Survey, *in vivo*
Data format	Raw
Experimental factors	Diabetic mellitus (DM) was induced by streptozocin (STZ). A novel polysaccharide was extracted from peach gum exudate, coded as PGPSD.
Experimental features	The water consumption of mice was traced during the courses of metformin hydrochloride and PGPSD treatments.
Data source location	Wuhan in China, 29°58′N–31°22′N, 113°41′E–115°05′E.
Data accessibility	The data is available with this article.

**Value of the data**

• The water consumption of DM mice could reflect the therapeutic effect of PGPSD polysaccharide.
• The data could be compared to other anti-diabetic polysaccharide studies.

## 1 Data

**Fig. 1:**
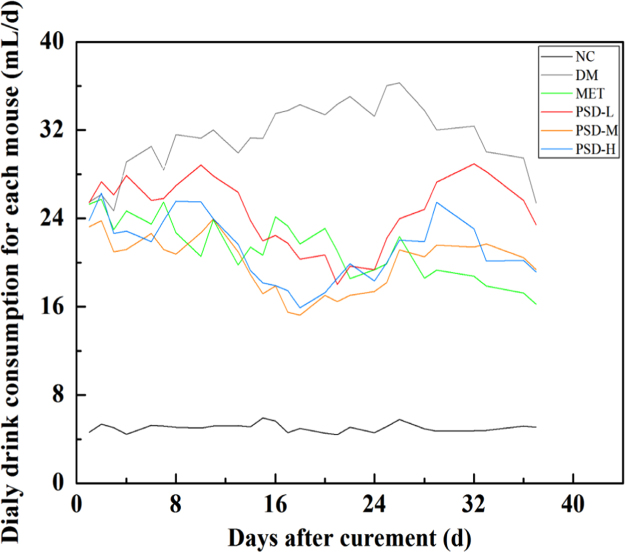
Average water consumption in diabetic mice administrated with PGPSD or metformin hydrochloride compared with normal mice administrated with sterile water.

The data exhibited the average water consumption of each DM mouse administrated with PGPSD polysaccharide or metformin hydrochloride for consecutive days. The daily water consumption of each group with different treatments was shown in Fig. 1.

## 2 Experimental design, materials and methods

### 2.1 The extraction of PGPSD polysaccharide

The peach gum sampled from fruit experiment field at Huazhong Agricultural University (Wuhan, in China) was dissolved in boiling water with the ratio of 1/100 (w/v) according to the method of Simas et al. [1], [2]. The concentrated solution was precipitated in ethanol overnight. The precipitated pellets were then de-proteinized following the Sevag procedure [3], decolorized using 30% hydrogen peroxide, dialyzed (MWCO 14,000) against distilled water for 2 d, and then frozen-dry to crude polysaccharide powder. The powder was re-dissolved and applied to DEAE-52 cellulose column, eluted with 0.5 mol/L NaCl solution. Fractions were collected, enriched, then dialyzed (MWCO 14,000) for 3 d, finally frozen-dried to the authentic polysaccharide (designated as PGPSD) [1].

### 2.2 Drug and PGPSD administration

Diabetic mellitus (DM) mice (male C57BL/6 mice) induced by streptozocin [4] were randomly divided into five groups with different treatments, namely, DM group (without any treatment), MET group (administrated with metformin hydrochloride at 200 mg/kg BW/day) and PSD-L/M/H group (administrated with PGPSD at 200/400/800 mg/kg BW/day, respectively) (*n*=7) [1], [5]. The intragastric administration lasted for 37 d, while the normal control mice (named NC group) were treated with same volume of sterile water. Water consumption was recorded daily. All experiments were conducted in the Public Health Service Policy on Use of Laboratory Animals in China.

## Funding sources

This study was supported by the National Natural Science Foundation of China (No. 31272120) and the China Agriculture Research System (No. CARS-31).
