# Supplementary material for: Data on water consumption in streptozotocin-induced diabetic mice by a novel peach gum-derived polysaccharide
Source: Data Brief. 2017 Apr 23;12:358–60. doi: 10.1016/j.dib.2017.04.022 (PMC5412007; doi:10.1016/j.dib.2017.04.022)
Supplement: Supplementary file 1 — Supplementary material [file mmc1.docx]

## Conflict of interest

## The authors declare there is no conflict of interest.
